# Flow Cytometry Sorting to Separate Viable Giant Viruses from Amoeba Co-culture Supernatants

**DOI:** 10.3389/fcimb.2016.00202

**Published:** 2017-01-06

**Authors:** Jacques Y. B. Khalil, Thierry Langlois, Julien Andreani, Jean-Marc Sorraing, Didier Raoult, Laurence Camoin, Bernard La Scola

**Affiliations:** ^1^Unité de Recherche sur les Maladies Infectieuses et Tropicales Emergentes, UM63 Centre national de la Recherche Scientifique 7278 IRD 198 Institut National de la Santé et de la Recherche Médicale U1095, Facultés de Médecine et de PharmacieMarseille, France; ^2^Institut Hospitalo-Universitaire Méditerranée Infection, Pôle des Maladies Infectieuses et Tropicales Clinique et Biologique, Fédération de Bactériologie-Hygiène-Virologie, Centre Hospitalo-Universitaire Timone, Assistance Publique–Hôpitaux de MarseilleMarseille, France; ^3^Becton Dickinson (Life Sciences–Biosciences) 94523Rungis Cedex, France

**Keywords:** giant viruses, flow cytometry, viral mixture, sorting, amoeba co-culture

## Abstract

Flow cytometry has contributed to virology but has faced many drawbacks concerning detection limits, due to the small size of viral particles. Nonetheless, giant viruses changed many concepts in the world of viruses, as a result of their size and hence opened up the possibility of using flow cytometry to study them. Recently, we developed a high throughput isolation of viruses using flow cytometry and protozoa co-culture. Consequently, isolating a viral mixture in the same sample became more common. Nevertheless, when one virus multiplies faster than others in the mixture, it is impossible to obtain a pure culture of the minority population. Here, we describe a robust sorting system, which can separate viable giant virus mixtures from supernatants. We tested three flow cytometry sorters by sorting artificial mixtures. Purity control was assessed by electron microscopy and molecular biology. As proof of concept, we applied the sorting system to a co-culture supernatant taken from a sample containing a viral mixture that we couldn't separate using end point dilution. In addition to isolating the quick-growing *Mimivirus*, we sorted and re-cultured a new, slow-growing virus, which we named “Cedratvirus.” The sorting assay presented in this paper is a powerful and versatile tool for separating viral populations from amoeba co-cultures and adding value to the new field of flow virometry.

## Introduction

Flow cytometry is a tool which is increasingly used in environmental microbiology (Veal et al., 2000; Vives-Rego et al., 2000; Brussaard et al., 2001; Bergquist et al., 2009; Wang et al., 2010) and biotechnology (Bergquist et al., 2009; Comas-Riu and Rius, 2009). In the virology field, flow cytometry plays a key role in both viral research and diagnostic laboratories (Wattré, 1992; McSharry, 1994; She et al., 2008). However, flow cytometry's contribution to the virosphere has a number of critical drawbacks, particularly in the research field where the main obstacle to the detection of viruses is their small size. Viruses are often less than 100 nm in diameter and therefore can barely be separated from the background. However, since the late 1990s, several reports have shown that viruses can be detected in various environmental samples using flow cytometry. For instance, Brussaard et al. managed to detect viral particles and were able to distinguish between different virus families based on their light scatter and green fluorescent intensity after staining with the nucleic acid specific dye SYBR® green I (Brussaard et al., 2000; Brussaard, 2004). Marie et al. reported that viruses could not only be detected, but also enumerated using flow cytometry after staining with SYBR® green I (Marie et al., 1999). We are recently witnessing the new emerging flow virometry technique, where Martínez Martínez et al. developed a new approach, that employs fluorescence-activated sorting and whole genome amplification to produce dsDNA-enriched libraries from discrete viral populations in water samples (Martínez Martínez et al., 2014). Nevertheless, the flow cytometers used are often specially developed and optimized for virus detection or enumeration (Nunez, 2001; Wang et al., 2010), and researchers have relatively limited access to these systems. Although these systems are efficient for the genomic analysis of viral populations they have never been used for further culture attempts. Since the first fluorescence-activated cell-sorting (FACS) technology description by Herzenberg in 1976 (Sweet and Herzenberg, 1976), flow cytometry cell sorters have developed the additional capability to allow physical separation of a heterogeneous mixture of biological particles for further analysis, such as downstream genomics or proteomics. While sorting studies and most single cell studies focused on many cell types, bacteria and cyanobacteria, viral sorting remained rare and difficult to execute, but nothing prevented scientists from developing robust and highly sophisticated cytometers to target viruses, like the works of Allen et al., Martinez-Martinez et al., Gaudin and Barteneva, as they represent the only actual studies presenting sorting of viruses and flow virometry (Allen et al., 2011; Martínez Martínez et al., 2014; Gaudin and Barteneva, 2015). Meanwhile, the discovery of giant viruses turned the virology era upside down and changed how we define a virus. Giant viruses broke the boundaries with the rest of the virosphere due to their particles and genomes sizes, ranging from 200 nm (Boyer et al., 2009; Colson et al., 2013b; Reteno et al., 2015) to more than 1 μm (La Scola et al., 2003; Philippe et al., 2013; Legendre et al., 2014) for their virion size, and from 350 kpb (Boyer et al., 2009; Reteno et al., 2015) to more than 2.4 Mpb for their genome size (Philippe et al., 2013). Viruses in general are ubiquitous and are the most numerous and diverse biological entities on our planet (Edwards and Rohwer, 2005). Giant viruses represent a large part of this phenomenon (Claverie, 2004; Colson et al., 2013a). Improved isolations strategies for these viruses have been proposed for almost 15 years (Khalil et al., 2016a) and since the beginning of this era, isolation by co-culture has shown mixtures of viruses in the same sample. Khalil et al. detected a number of giant viral sub-populations and mixtures in samples (Khalil et al., 2016b). Melbournevirus (Doutre et al., 2014) was isolated from the same sample that also enabled the isolation of *Pandoravirus dulcis* (Philippe et al., 2013). *Pithovirus sibericum* and *Mollivirus sibericum* were also recovered from the same sample (Legendre et al., 2014, 2015). These mixtures are difficult to deal with, particularly at the cloning stage of the sequencing process. For example, we had to address contamination by other giant viruses, which we were unable to detect during the cloning procedures, although their genomes were detected through the analysis of genomic data (unpublished data). Moreover, some authors recently showed that viral particles could be highly damaged by the sorting process (Martínez Martínez et al., 2014). In this study, we propose a new approach for sorting giant viruses from amoeba co-culture supernatants containing mixtures of viruses using flow cytometry cell sorters. After showing that the system is able to separate artificial mixtures of giant viruses, we applied our approach to a natural mix of viruses observed in an amoeba co-culture supernatant drawn from a water sample. The procedure enabled us to provide pure clones of two viruses, including one new species, which we described this later at both the genomic and infectious cycle levels (Andreani et al., 2016).

## Materials and methods

### Artificial mixtures of viruses

We followed the same strategy reported in our previous work to treat and stain the microorganisms before using flow cytometry for sorting (Khalil et al., 2016b). Briefly, we used the following purified virus strains: *Acanthamoeba polyphaga Mimivirus* (APMV) (La Scola et al., 2003; Raoult et al., 2004), Marseillevirus (Boyer et al., 2009), Faustovirus (Reteno et al., 2015), and a new Pandoravirus which was recently isolated in our lab (Dornas et al., 2015). Three preparation conditions were tested: in the first assay, all particles were heated to 80°C and SYBR® green stained for 10 min (SYBR® green I nucleic acid gel stain; molecular probes, Life Technologies USA) with a final concentration of 10^−4^ dilution of the commercial stock solution. In the second condition, another category of the same organisms was fixed with PFA 4% (Paraformaldehyde, Sigma, France) before SYBR® green staining. Finally, in the third condition, viruses were directly stained using SYBR® green staining following overnight culture at room temperature. We then prepared mixtures of different viral populations at various concentrations. We took two distant concentrations of viruses, with 10^4^ virus/ml as the lowest range and 10^8^ viruses/ml as the highest range, in order to frame the viral concentrations that we often find in the culture supernatant and to check the detection and sorting limits.

### Flow cytometry viral sorting procedure and analysis

Megamix Plus SSC beads and Megamix Plus FSC beads (Biocytex, Marseille, France) were used to calibrate the cytometer settings. Each viral population was assessed first in order to gate populations corresponding to the pure viral fraction. Then we proceeded to analyze and sort mixed fractions. We tested three sorters as detailed below. A BD FACSAria™ Fusion Special Order (SORP) cell sorter cytometer (BD Biosciences) equipped with a combination of six lasers was used: 355 (15 mW), 405 (85 mW), 488 (50 mW), 561 (50 mW), and 640 nm (100 mW). A photodiode was used as a standard FSC-detector on this instrument. Sorting was performed using a 70 μm nozzle, pressure of 70 PSI and a frequency of 87 kHz. A BD FACSJazz™ cell sorter (BD Biosciences) equipped with a combination of three lasers was also used: 405 (50 mW), 488 (80 mW) and 561 (75 mW). As a standard FSC-detector on this instrument, a photomultiplier tube was used. Sorting was performed using a 100 μm nozzle, pressure of 27 PSI and a frequency of 39 kHz. A BD Influx™ cell sorter (BD Biosciences) equipped with a combination of five lasers was used: 355 (100 mW), 405 (50 mW), 488 (200 mW), 561 (75 mW), and 640 nm (50 mW). This later instrument was equipped with two photomultiplier tubes for the polarized light detection (Brewster mirror) (FSC Per: 90° light, and FSC Par: 180° light), and with the small particles option consisting of 20x, 0.42NA microscope objective mirror pinhole, a pinhole camera, and an obscuration bar allowing a collection angle ranging from 14 to 30°. Sorting was performed using a 70 μm nozzle, pressure of 60 PSI and a frequency of 100 kHz. All data were analyzed using FlowJo v10 (Tree Star). For the three instruments described, triggering was based on the fluorescence channel for SYBR® green detection for the smallest particles (on the 488 nm laser with 530/30 BP filter for the BD FACSAria™ Fusion, 513/17 BP filter for the BD FACSJazz™, 530/40 BP filter for the BD Influx™). We applied the same protocol and procedure to the three tested sorters. All the sorting experiments were carried out using sorters in a biosafety cabinet for the best safety performance and sorting results. Identification of the different virus populations was performed using a crossing-gate to compare bi-parametric profiles FSC vs. SSC, and SSC vs. SYBR® Green. The flow sheath was 0.22 μm in-line filtered. The pressure was allowed to stabilize for at least an hour before starting experiments. For analysis of the sorted fraction's purity, collected samples were analyzed under the same conditions. The purity calculation was based on the ratio between the different population counts, by applying the following equation: [% Sorted fraction = (#*Sorted fraction /# all fractions*) x 100] after reanalysis of the sorted population. All experiments and controls were realized in triplicate.

### PCR analysis and transmission electron microscopy for purity control

The results of the sorting purity were confirmed by an in-house designed PCR system targeting the different giant viruses used in this work (Table 1). PCR was directly performed on each sorted fraction. The real time PCR assay protocol is detailed in the works of Ngounga et al. (2013). Sorting purity was also confirmed by negative staining electron microscopy. Briefly, 10 μl of the fixed sorted viral suspension was deposited on the carbon grids for 10 min following the glow discharge, so that it adhered to the grids. It was then contrasted using a 1% solution of ammonium molybdate. Images were collected using a Tecnai G2, operating at 200 keV (FEI Germany). We counted 1000 particles per grid.

**Table 1:** **The PCR system used for post sorting purity check**.

**Virus**	**Target gene**	**Sequence (5′ to 3′)**
Mimivirus A	R651 hypothetical protein	F: TGGATACATTGATGGTTGATAA
		R: TTTCGACTTTACACTTGGGATTG
		P: TTATGAAAAACCTAATCCAGAAGATT
Marseillevirus	Intergenic region	F: TCTGGGAGTGGGCTTTATCT
		R: AGGGTAATGACCTCGGGTA
		P: AGGATTGAACCTTCGCTGTTAC
Faustovirus	RNA polymerase II rpb1	F: CAAAGGCTATTGAGGCGATTTG
		R: ATGATTGTGCTGCTAGGATACC
		P: CGTTACACCAACGCTTTAATGGCGC
Pandoravirus	RNA polymerase II rpb2	F: ACGTGCGAGTATTTGCTGTT
		R: GTGTCGCAGATCAAGATGC
		P: GACGCACGCGTGGTTGAT

### Validation of the assay by sorting an amoeba co-culture supernatant containing a viral mixture

We applied our sorting procedure to a co-culture supernatant of an environmental water sample inoculated on *Acanthamoeba castellanii*, and contained two viruses, that we failed to clone by end point dilution (Lynn, 1992). Our negative control as a background noise of the medium was the supernatant of an uninfected amoebal culture. We used the BD FACSJazz™ sorter under the conditions described above. We used a double strategy of sorting, strating with a first sort in a one drop enrichment technique, to recover the minority fraction of the unidentified virus, followed by the one drop pure sort technique, to get the most pure fraction of this later. Briefly, the one-drop enrichment strategy consists of a positive sort decision of one drop containing one or more targeted particles without taking care of the purity (presence of non-target particles is allowed), for this, the efficiency is not affected, and so all targeted particles are sorted, but the sorted fraction is not pure. As for the One-drop pure strategy, it consists of a positive sort decision of one drop containing one or more target particles without non-target particles inside and in the adjacent regions of the previous and following drops (coincidence mask). The efficiency is affected due to the sorting aborts decision of the flow cytometer, and the percentage of sorted target particle is not 100% (Poisson statistics dependent), so the sorted fraction should be pure.

**Figure 1:**
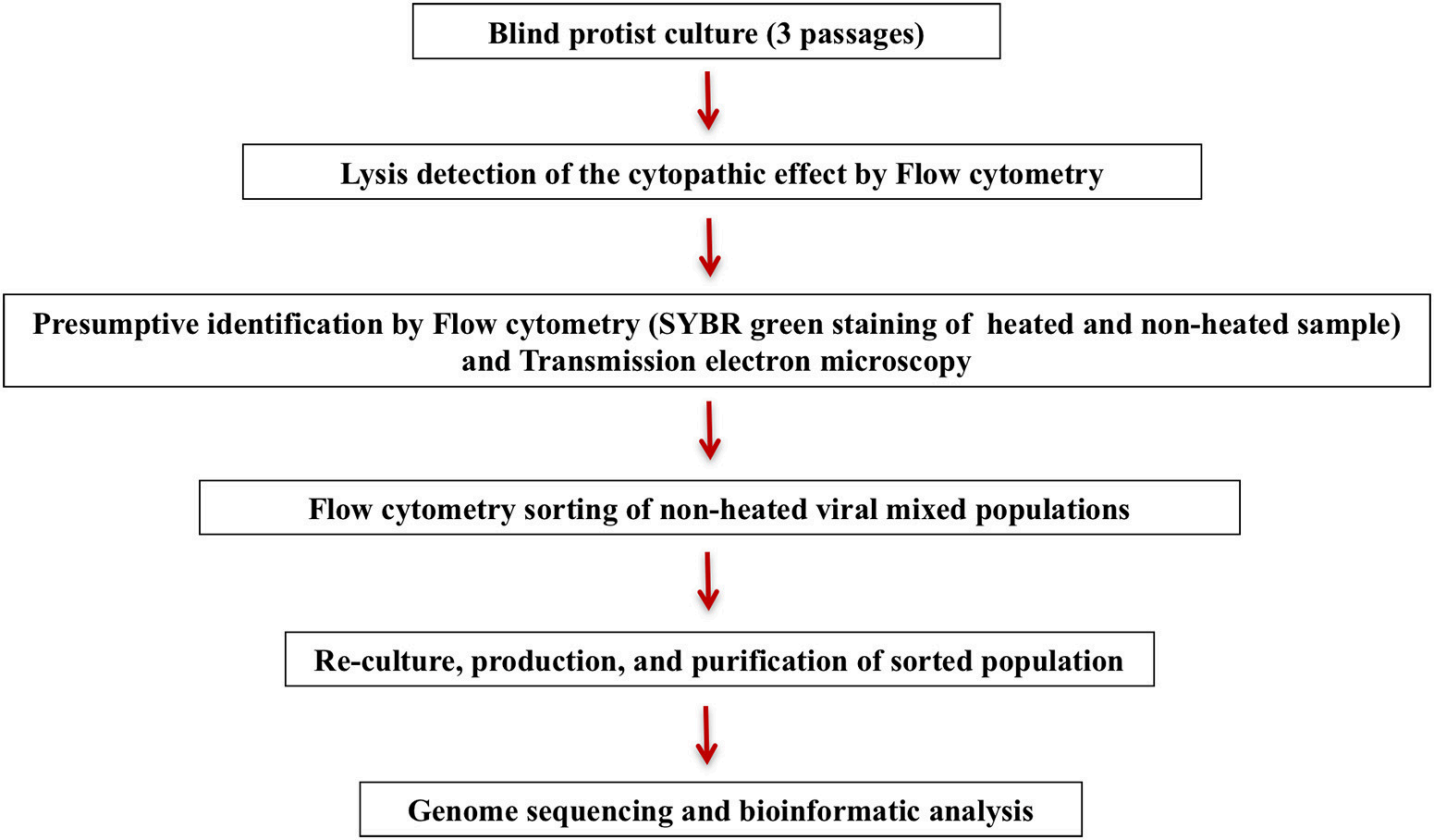
**Representation of giant virus isolation and sorting strategy**.

After sorting, the fraction containing the unidentified virus was inoculated onto a fresh amoeba monolayer in a 24-well plate, and sub-cultured twice after detecting a cytopathic effect. Following the two subculture steps, the amoeba supernatant was passed on to flow cytometry and transmission electron microscopy for purity check, and was then produced in large volumes for genome sequencing with MiSeq Technology (Illumina, Inc., San Diego, CA) using the paired-end and mate-pair applications in parallel in a 2 × 251 bp run for each bar-coded library. This entire procedure from culture till genome sequencing is summarized in Figure 1.

## Results

### Flow virometry sorting of giant viruses

**Figure 2:**
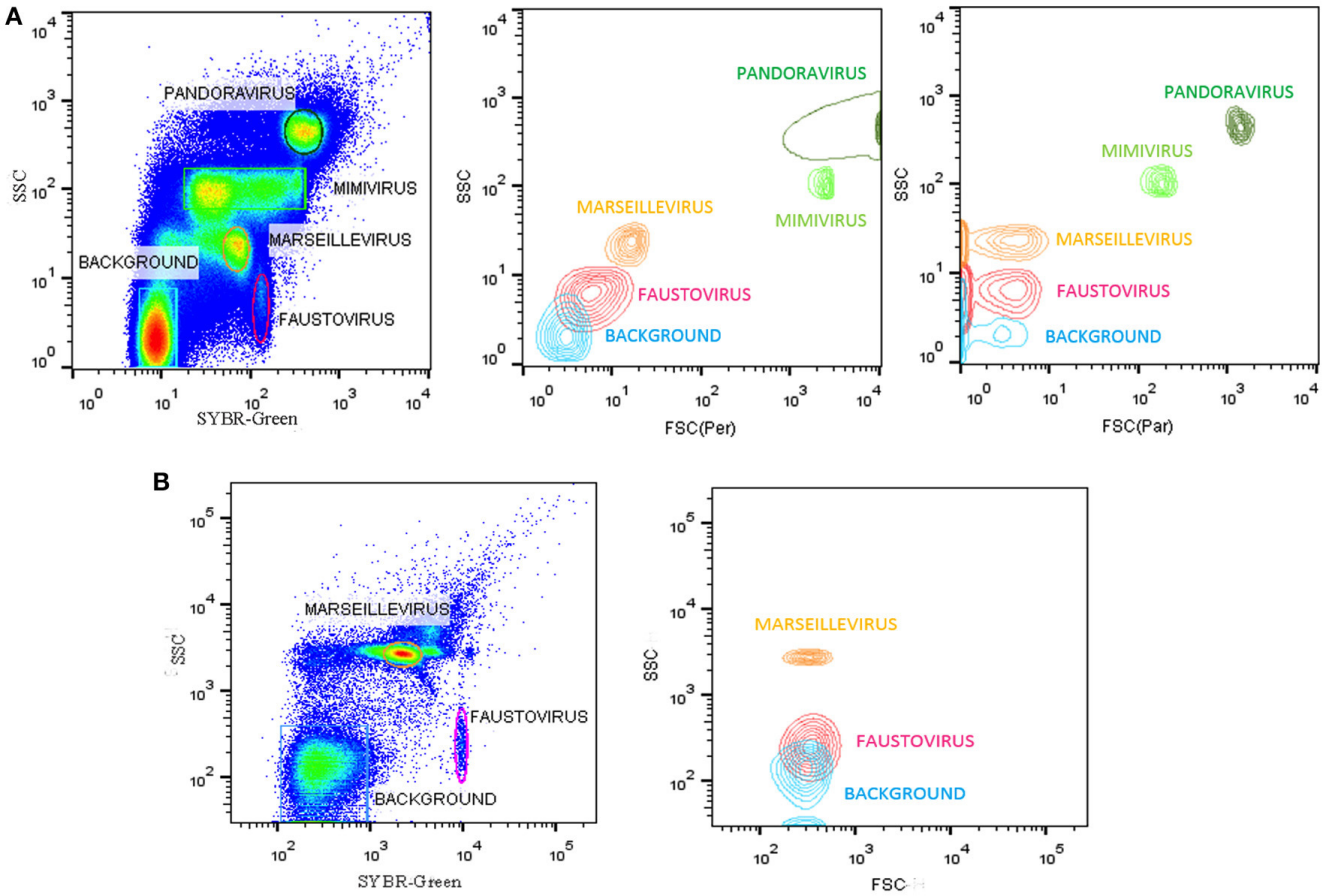
**(A)** Representative FACS plots obtained with the BD Influx™, analyzed by FlowJo, displaying live viral population stained by SYBR® green for an overnight at room temperature in the tested mix for the sorting in the gated plots SYBR® green vs. SSC. The plots FSC (Per) vs. SSC, and FSC (Par) vs. SSC are the projections of the gated populations in the plot SYBR green vs. SSC. FSC (Per) and FSC (Par) are both forward scatter parameters allowing polarized light collection through a transparent dielectric surface. FSC (Per) has been adjusted to be the most sensitive parameter allowing good discrimination between particles sizes and composition in e.g., Faustovirus and Marseillevirus, which is not possible with FSC (Par), and the biggest particles are stacked on the last channel because of a very different size compared to the smallest particles (200 nm vs 1 μm). FSC (Par) is less sensitive and so we are able to detect smallest and biggest particle on the same scale, which is not possible with FSC (Per). **(B)** Representative FACS plots obtained with the BD FACSAria™ Fusion and analyzed by FlowJo displaying the mix of Faustovirus and Marseillevirus in the gated plots SYBR® green vs. SSC. Plot FSC vs. SSC is the projections of the gated populations in the plot SYBR green vs. SSC.

**Figure 3:**
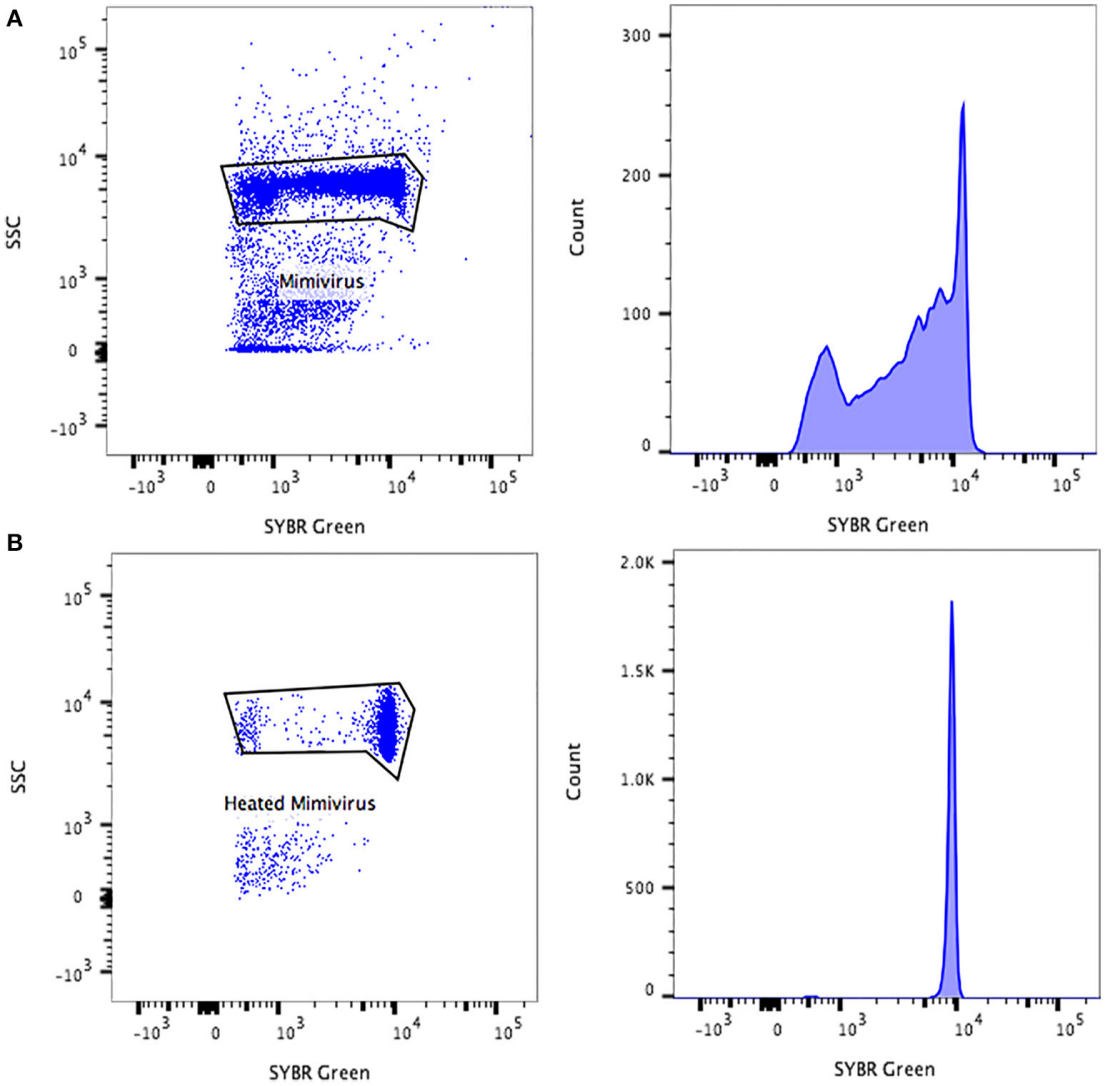
**Representative FACS plots and fluorescence histograms analyzed by FlowJo, displaying the Mimivirus viral populations (A)** non-heated, **(B)** heated.

All flow cytometry populations were gated initially on the plots of SYBR® green vs. SSC and FSC vs. SSC (Figure 2). The gating frames were kept fixed throughout the measurements. The forward scatter, side scatter, and SYBR® green-positive gated populations were always clearly distinguishable, particularly for Pandoravirus (Philippe et al., 2013), Mimivirus (La Scola et al., 2003; Raoult et al., 2004), and Marseillevirus (Boyer et al., 2009) allowing for an easy sorting purification mode. For Marseillevirus (Boyer et al., 2009) and Faustovirus (Reteno et al., 2015), gating and sorting was only possible using the SYBR® green stain. Indeed, it was difficult to differentiate these two viruses on the FSC vs. SSC plots since these viruses are almost the same size (respectively 200 and 197 nm). The detection limit of all tested dilutions resulted from the detection limit of the flow cytometers, where it was around 10^2^ particles/ml. We observed that heating between 70° and 80°C for 5 min improved the sharpness of the fluorescence profiles on the dot plot especially for Mimivirus, but no significant difference regarding the fluorescence intensity could be observed (Figure 3).

### Purity of sorted samples

**Figure 4:**
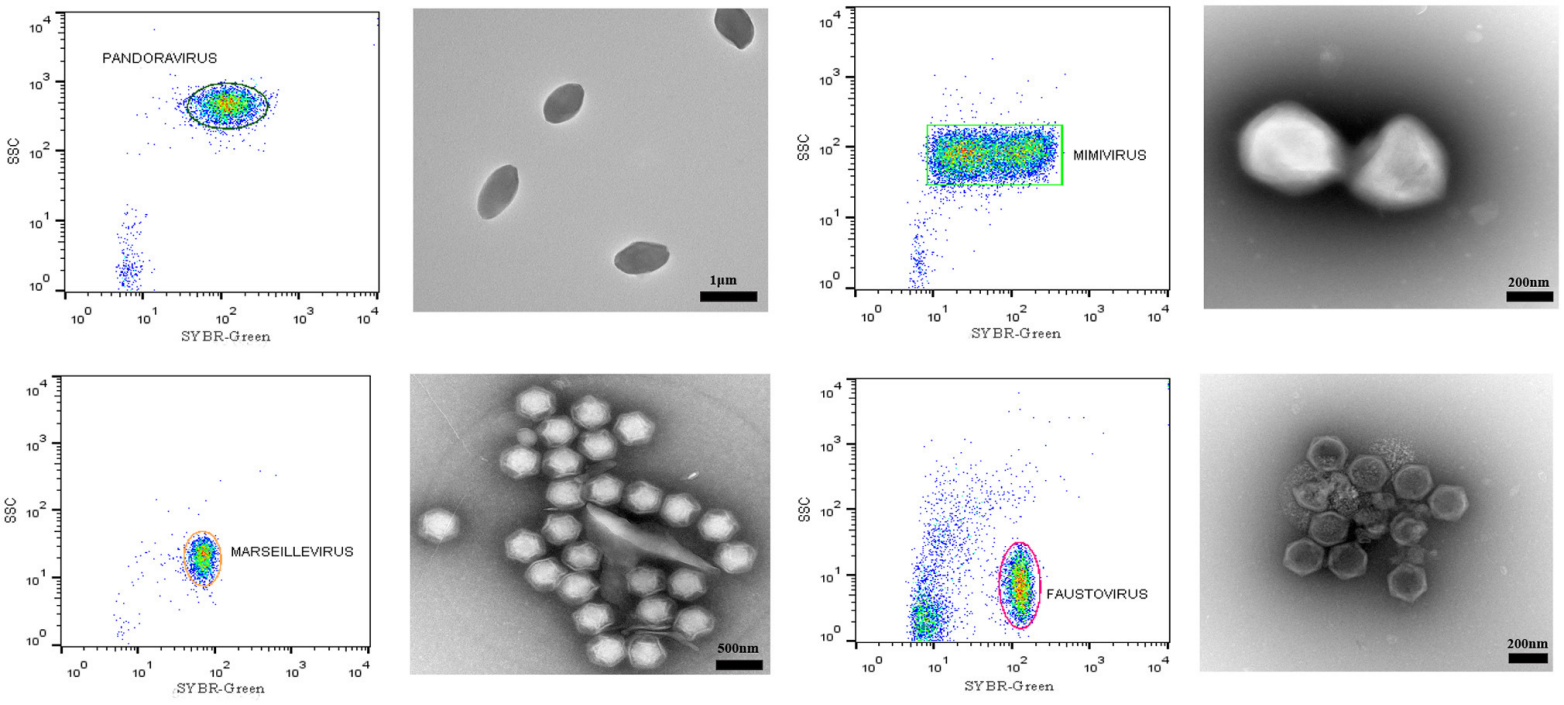
**Representative FACS plots obtained with the BD Influx™ and the BD FACSAria™ Fusion, analyzed by FlowJo**. These are representative for all three tested sorters and display the results of each sorted population. The percentage of purity for each fraction was more than 99%. Transmission electron micrographs of each sorted viral population confirmed the purity of the samples.

**Figure 5:**
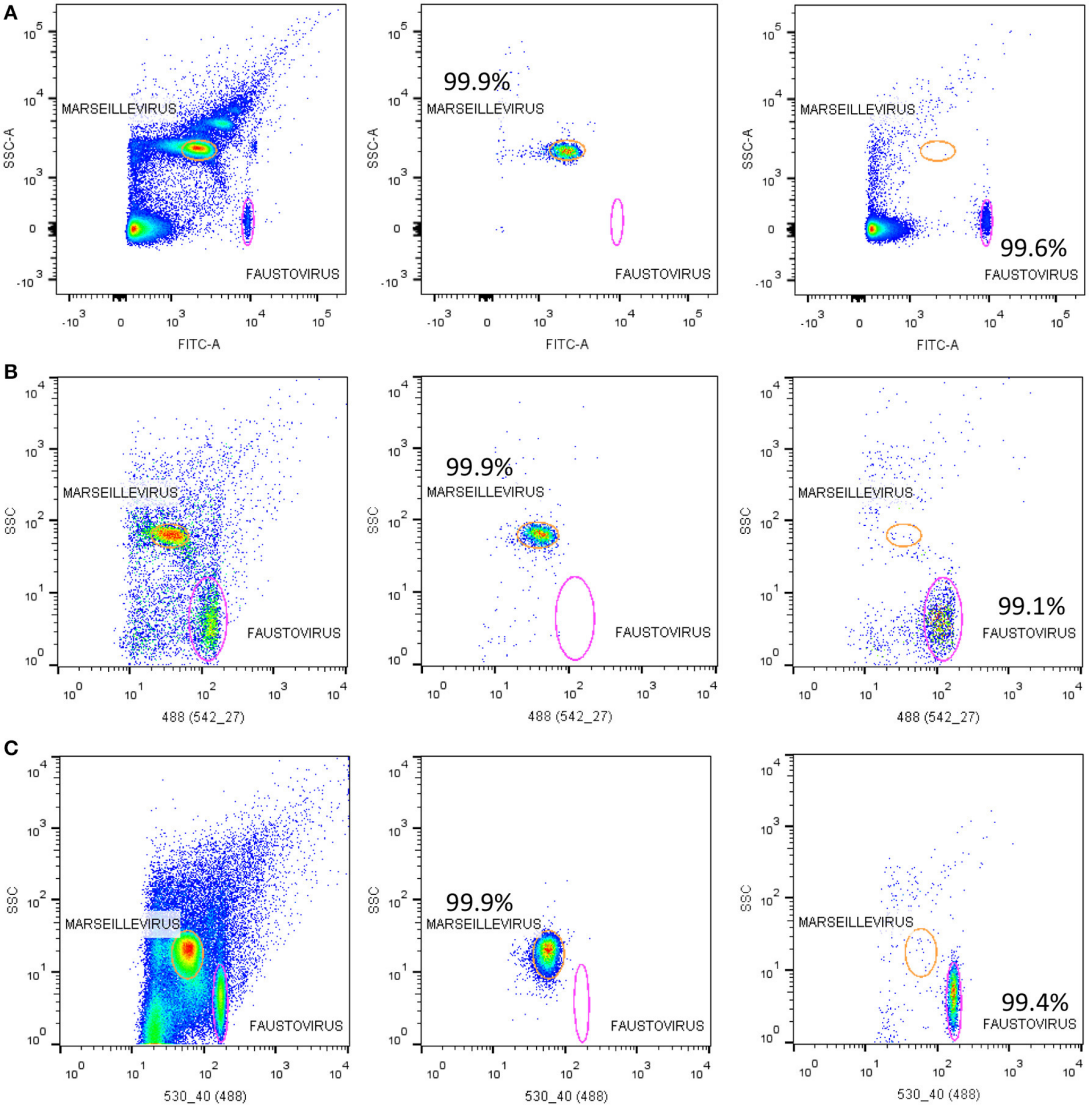
**Representative FACS plots analyzed by FlowJo**. Showing the results of sorting samples on the three tested sorters; **(A)** the BD FACSAria™ Fusion, **(B)** the BD FACS Jazz™, and **(C)** the BD Influx™. The sorting purity results of each viral population of Marseillevirus and Faustovirus are also displayed.

On average, the purity of the sorted viral populations extrapolated by the machine after the post-sorting analysis control was more than 99% for all viruses tested on all three sorters. Reanalysis of the sorted samples of viruses by flow cytometry revealed a distinct population corresponding to one unique population in the gated area for each virus (Figure 4). Our restrictive sort gates eliminated the risk of cross sorting. Martínez Martínez et al. noticed a damaged capsid on TEM analysis which may have been due to the sorting procedure (Martínez Martínez et al., 2014). It should be added that this may also be due to the treatment of samples before sorting. The sorted population did not appear to be damaged by the sorting procedure, with the exception of some Faustovirus particles (Figure 4). Moreover, the non-treated viral particles may possibly be robust or more resistant to the sorting procedure. The time needed to sort the mixtures of viral populations varied proportionally with the concentration and the percentage of viruses in the starting sample, together with the event rate during the sorting process. The electron microscopy carried out on the sorted populations confirmed the results of the sorting purity and no cross-contaminated populations could be detected on the 1000 counted particles per grid for each sorted fraction. PCR detection also confirmed the purity of the samples (Table 2). Comparable results of gating strategies, sorting and purity were obtained on the three used flow cytometer cell sorters (Figure 5).

**Table 2:** **The PCR detection results for the post sorting purity check**.

**Sorted fraction**	**qPCR Ct mean (*n* = 4)**			
	**Pandoravirus**	**Mimivirus**	**Marseillevirus**	**Faustovirus**
Pandoravirus	21.23	No detection	No detection	No detection
Mimivirus	No detection	22.20	No detection	No detection
Marseillevirus	No detection	No detection	17.97	No detection
Faustovirus	No detection	No detection	No detection	21.39

### Validation of the assay by sorting an amoeba co-culture supernatant containing a viral mixture

**Figure 6:**
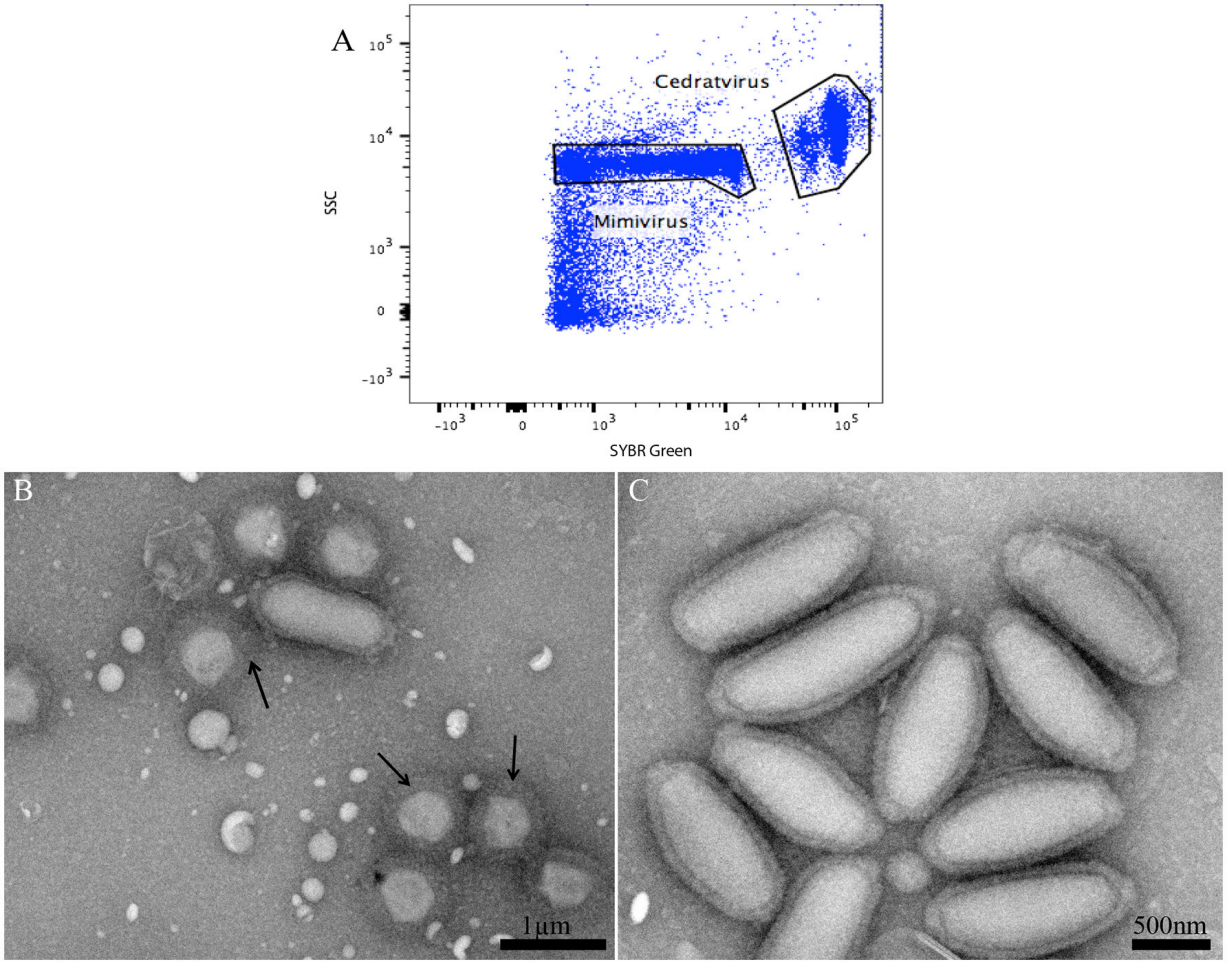
**(A)** Representative FACS plots obtained with the BD FACS Jazz™, analyzed by FlowJo, displaying the two viral populations isolated from the water sample. **(B)** Transmission electron microscopy of the sample containing the viral mixture displaying the new isolated oval shaped Cedratvirus with its 1.2 μm medium size. The presence of the 650 nm sized Mimivirus, in its typical icosahedral capsid shape with fibrils (arrows), is also noted. Particle size is measured using imageJ software. **(C)** Transmission Electron Microscopy of the sorted population of Cedratvirus for purity control.

In the course of our strategy for high throughput virus isolation, an environmental water sample inoculated on *Acanthamoeba castellanii* led to amoeba lysis. According to our previous techniques (Khalil et al., 2016b), (Figure 1), the first flow cytometry identifications realized on the amoeba co-culture supernatant revealed the presence of a viral mixture, where we had a population with the typical gate aspect and fluorescence profile of Mimivirus (confirmed by PCR identification), and a new profile, undetected previously, and corresponding to a higher SSC and greater fluorescent intensity (Figure 6A). Electron microscopy confirmed the presence of oval shaped viral particles mixed with Mimivirus (Figure 6B). This new unidentified population presented two distinct subpopulations, but after heating and staining, we only had one population well defined. Moreover, the Mimivirus population was richer than the unidentified population, where around 70% of Mimivirus and 30% of the new population was found on all acquired events. Due to the fast viral fitness or the multiplication speed of Mimivirus, all attempts to separate the two viruses by end point dilution (Lynn, 1992) only led to the isolation of the Mimivirus in pure culture, and serial subculture led to the loss of the unidentified virus. In details, we did a TCID 50 going from 10^−1^ to 10^−13^, the cytopathic effect could be observed at 10^8^ and 10^9^ for only Mimivirus while at 10^7^ we had the mix Mimivirus, and the new viral population, which was easily observable under light microscopy for its big particles sizes. As we mentioned before, we had two subpopulations in the gate of the new viral population, but these subpopulations became one after heating, so we sorted these subpopulations as one. The sorting by one-drop enrichment strategy, followed by one-drop pure sorting strategy allowed us to separate and purify this new viral population (Figure 6C). In addition, and after sorting, we controlled again by an end point dilution and monitored the cythopatic effect where we only had the biggest particles and no Mimivirus could be detected. The pure culture of the sorted population was also processed through transmission electron microscopy, confirming that this new giant virus isolate had a shape close to that of *Pithovirus sibericum* (Legendre et al., 2014) but with new structural features. After production and genome sequencing, we described our new “Cedratvirus,” that we added to the family of giant viruses (Andreani et al., 2016).

## Discussion

In this work, we continued to apply flow cytometry to the isolation of giant viruses through co-culture on amoeba (Khalil et al., 2016a,b), by developing a simple, robust sorting system capable of purifying a viable viral mixture with the highest purity and speed in comparison with previously tedious end point dilution systems. This will provide pure clones of viable viruses, which will offer suitable reference genomes to facilitate the interpretation of viral genomic and metagenomic data for a better understanding of the evolution, adaptation, ecology, and potential pathogenicity of viruses. The accurate analysis of viruses in the environment using commercial flow cytometry instruments remains a challenge (Wang et al., 2010). Flow cytometric methods have been optimized (Brussaard, 2004) and used on natural viral populations for enumeration purposes (Marie et al., 1999) and genomic analysis (Martínez Martínez et al., 2014). Therefore, for this study, flow cytometry was used to sort a mixed viral assembly consisting of four known giant viruses. At the same time, other studies have used more sophisticated flow cytometry cell sorters to push the limits in terms of detecting and sorting single virus particles (Allen et al., 2011), even the smallest of them (Gaudin and Barteneva, 2015). Polarized light detection and a small particles option detector (wider obscuration bar and magnification lens) were very sensitive in terms of scatter detection. In contrast, we demonstrate that, without the use of any special equipment and by using standard flow cytometry instruments working with sense-in-air or sense-in-quartz systems, we were able to define the profiles of viral particles ranging from 197 nm to 1 μm and sort them by side scatter and DNA content (SYBR green). Once again, we found a correlation between the fluorescence signal and viral genome size (Khalil et al., 2016b) but, no linear relationship could be found, this could be possibly due to the viral genome or DNA compaction, and or to the capsid composition; thus, preventing the detection of the real fluorescence signals. By using SYBR® green intensity we were able to detect the smallest viral population in the mixture, and discriminate it from the background. Moreover, based on SSC profile and genome size with SYBR® green staining, we were able to discriminate and sort two populations, one measuring 200 nm and the other 197 nm. These two populations present two different FSC vs. SSC profiles, suggesting differences in the composition or ultrastructure of the virus. Live viruses may have a structure that prevents the access of SYBR® green to nucleic acids, to this we add the possibility of the DNA compaction that could also prevent the stain access or uptake. Detergent or heat treatment up to 95°C may be needed to denature the virus capsid and allow the stain to penetrate (Brussaard, 2004) but we still have the DNA compaction that can prevent the establishment of a linear correlation between DNA content and fluorescence signals. In our case, for virus material and for a fixed concentration of dye, the effect of heating was assessed. In general, we observed that heating between 70° and 80°C for 5 min improved the sharpness of the fluorescence profiles on the dot plot but resulted in no significant difference to or shifts in the fluorescence. Furthermore, the genome of Cedratvirus (Andreani et al., 2016) is smaller than the one of Mimivirus but still has higher fluorescence than this latter. By this we couldn't find again a linear correlation between genome contents and SYBR® green signals. We postulate, that in our case, the fluorescence profiles are the sum of the SYBR® green uptake by the capsid and what was stained from the DNA contents, and so together, the capsid composition of Cedratvirus and the lower compaction of its genome regarding the ones of Mimivirus may allow a better diffusion of the SYBR® green. However, as our goal was to sub-cultivate our sorted viruses, it would not be suitable to heat the samples before sorting as flow cytometry analyses without heating has the same results. In our case, therefore, samples did not require any pretreatment. The sorting of live viruses in order to re-culture these latters is in itself a big challenge for the purity control, and a rare remarkable work, that deserves to emphasize on it along with the study by Gaudin and Barteneva for being the only other one that managed to do it (Gaudin and Barteneva, 2015). We are aware of the detection limits of this technique, as, even given significant progress in terms of equipment, the light-scatter responses of the sub-micron particles measured are close to the limits of size detection. For the Faustovirus we observed a loss of the fluorescence after sorting, so we managed to stain the virus again with the SYBR® green in order to check the purity, as this phenomenon could be either due to the sorting process or to the Faustovirus viral capsid (Klose et al., 2016). When we compared the post sorting data and the purity control, more background in the case of the Faustovirus post sorting fraction was observed regarding to the other viruses. We can correlate this to the SYBR® green addition before and after the sorting process, which could generate more background noise. So for the preparation of the sample we can expect an improvement of the staining process to get better detection sensitivity. We also know that FSC is historically correlated to relative size and SSC to relative granularity, but this is dependent upon multiple parameters, particularly in the case of biological particles. As such, the membrane composition, refractive index of different biological compartments and, thus, the internal complexity will have an impact on the FSC and SSC signals. Hence, it is very complicated to directly match the FSC to the size between two particles, which do not have the same structure or material composition. The resolution for the FSC signals could range from 0.2 to 0.5 μm depending on many parameters, such as electronic noise, filtering the sheath fluid (0.1 vs 0.22 μm), PMT vs. photodiode detector, or laser power. Since the SSC is collected at 90° from the laser beam, the part of the optical background is limited (compared to the FSC). Theoretically, SSC is more sensitive than FSC but is less impacted by the size of the particle than the FSC parameter. However, in the case of viruses, the structural complexity of the particles (DNA, capsid and envelope) can be taken into account in the analysis in order to achieve better discrimination between viruses on the SSC parameter. Finally, to ensure a low detection limit, it would be reasonable to trigger a fluorescence parameter to capture a specific signal (with no laser wavelength going to the detector). Moreover, using a sorter instrument, the optical background for the FSC or SSC signals could be directly impacted by the sort setup. Indeed, the direct diffraction of the incident light, sheath pressure and height of the break-off point could generate more or less optical background due to vibration waves from the piezoelectric system. Sorters working with a sense-in-quartz system (where the interrogation point is in a cuvette before stream-in-air) are less impacted by these sort setups than instruments working with sense-in-air systems. For flow cytometry cell sorting, triggering the signal processing based on a threshold setting is not a trivial matter, particularly for a small particle mixture. An FSC or SSC threshold could be dangerous for the sorting results because of the contamination in the droplets by particles smaller than the threshold. What is above the threshold is not analyzed, and thus is not taken into consideration for the sort decision. This is why it would be better, if possible, to choose a generic biological stain for triggering, which would enable us to detect all biological particles. Flow cytometry is faster and more accurate than any other method currently used for the direct detection, quantitation and purification of virus particles. In the case of a mixed population, flow cytometry is still the best tool to obtain information on the abundance of one specific type of virus compared to other methods, such as electron microscopy or end point dilutions, which are time-consuming. The use of flow cytometry to discriminate and sort the mixtures of viruses encountered in culture and isolation processes added speed to the purification and cloning processes in order to get the best samples for genomic and proteomic data. Flow cytometry sorters' evolutionary technologies, amplified signals and increased sensitivity in order to achieve better results in terms of detection and sorting of small particles. Given these changes and the size of giant viruses, flow cytometry undoubtedly remains the most valuable tool in environmental virology, at least for the domain of giant viruses.

## Author contributions

JK, DR, LC, and BL designed research; JK, TL, JS, JA, and LC performed research; JK, TL, LC, JS, JA, DR, and BL analyzed data; JK, TL, LC, DR, and BL wrote the paper.

### Conflict of interest statement

The authors declare that the research was conducted in the absence of any commercial or financial relationships that could be construed as a potential conflict of interest.
